# Long-Term Persistence of Functional Thymic Epithelial Progenitor Cells *In Vivo* under Conditions of Low FOXN1 Expression

**DOI:** 10.1371/journal.pone.0114842

**Published:** 2014-12-22

**Authors:** Xin Jin, Craig S. Nowell, Svetlana Ulyanchenko, Frances H. Stenhouse, C. Clare Blackburn

**Affiliations:** MRC Centre for Regenerative Medicine, Institute for Stem Cell Research, School of Biological Sciences, University of Edinburgh, SCRM Building, 5 Little France Drive, Edinburgh, EH16 4UU, United Kingdom; University of Tokyo, Japan

## Abstract

Normal thymus function reflects interactions between developing T-cells and several thymic stroma cell types. Within the stroma, key functions reside in the distinct cortical and medullary thymic epithelial cell (TEC) types. It has been demonstrated that, during organogenesis, all TECs can be derived from a common thymic epithelial progenitor cell (TEPC). The properties of this common progenitor are thus of interest. Differentiation of both cTEC and mTEC depends on the epithelial-specific transcription factor FOXN1, although formation of the common TEPC from which the TEC lineage originates does not require FOXN1. Here, we have used a revertible severely hypomorphic allele of *Foxn1, Foxn1^R^*, to test the stability of the common TEPC *in vivo*. By reactivating *Foxn1* expression postnatally in *Foxn1^R^* ^/−^ mice we demonstrate that functional TEPCs can persist in the thymic rudiment until at least 6 months of age, and retain the potential to give rise to both cortical and medullary thymic epithelial cells (cTECs and mTECs). These data demonstrate that the TEPC-state is remarkably stable *in vivo* under conditions of low *Foxn1* expression, suggesting that manipulation of FOXN1 activity may prove a valuable method for long term maintenance of TEPC *in vitro*.

## Introduction

The thymus is the obligate site of T-cell development and is thus crucial for establishment of the adaptive immune system [1]. The epithelial compartment of the thymic stroma provides specialist functions required to mediate T cell differentiation and repertoire selection, and is broadly divided into two compartments, the cortex and medulla. The functional dichotomy between these compartments reflects functional differences between cortical and medullary thymic epithelial cells (cTEC and mTEC respectively). However, these TEC sub-lineages have a single origin in the endoderm of the third pharyngeal pouch, and clonal analyses have demonstrated the existence of a common TEPC within the population of cells that founds the TEC lineages [2]–[4].

FOXN1, a member of the forkhead family of transcription factors, is the gene mutated in the classical *nude* mouse, which exhibits congenital athymia and hairlessness [5]. In *Foxn1* null mice, the thymic primordium forms normally and expresses markers that, within the pharyngeal endoderm, are specifically associated with the thymic epithelial lineage. Thus, FOXN1 is not required for thymic epithelial lineage specification [3]. Neonatal activation of a revertible *Foxn1*-null allele resulted in generation of functional mini-thymi, indicating that up-regulation of FOXN1 in specified TEPC was sufficient to elaborate the TEC differentiation programme and further demonstrating that, in the absence of FOXN1, TEPC can remain functional for at least 3 weeks after FOXN1 expression is normally initiated [6]. Whether functional TEPC phenotype cells persisted beyond postnatal day 14 was, however, not tested.

We recently reported that FOXN1 is required for differentiation throughout lineage progression in both cTEC and mTEC, from exit from the undifferentiated TEPC state to terminal differentiation [7]. This conclusion was based on analysis of an allelic series generated with a novel revertible hypomorphic allele of *Foxn1*, *Foxn1^R^*, in conjunction with *FOXN1^−^* and wild-type (WT) alleles.

The *Foxn1^R^* allele was generated by knocking a loxP-flanked cassette containing the SV40 T antigen cDNA followed by a strong transcriptional stop element into intron 1b of the *Foxn1* locus. As previously described, this generated a revertible severely hypomorphic allele of *Foxn1,* which expresses 15% of wild-type levels of Foxn1 mRNA [7]. *Foxn1^R/−^* mice, which carry one revertible hypomorphic allele and one null allele of *Foxn1*, are functionally athymic. Indeed, the thymic phenotype of these mice is very similar to that of *Foxn1^−/−^* mice, in that the thymic primordium forms but never becomes colonized by haematopoietic or endothelial progenitors, and thus never supports T cell development [7]. However, due to the very low level of FOXN1 expression, evidence of initiation of the first events of the differentiation programme is observed in *Foxn1^R/−^* TEPC [7]. We and others have previously shown that blockade of Foxn1 mRNA expression results in developmental arrest of TEPC, and that postnatal reversion of the *Foxn1* expression blockade results in generation of functional and organised thymus tissue [6], [7]. However, the capacity of these arrested progenitors to persist long-term *in vivo* has not been tested. This question is of interest for strategies aiming to propagate TEPC long-term *in vitro* or to derive such cells from pluripotent or other cell types, since such cells are predicted to express low levels or no *Foxn1.* In some cell lineages the absence of transcription factors which promote lineage differentiation is known to result in fate shifting or loss of potency – as evidenced for instance by the altered identity of B cell progenitors lacking expression of *Pax5* [8] *-* and therefore the effect of long-term absence of *Foxn1* expression in cells initially specified as TEPC is not known.

Here, we have used the *Foxn1^R/−^* model to test the longevity of maturationally arrested TEPC *in vivo.* We show by analysis of spontaneous reversion of the *Foxn1^R^* allele in *Foxn1^R/−^; R26^CreERt2^* mice, that such TEPC can persist *in vivo* for at least 6 months.

## Results

### Reversion of the *Foxn1^R^* allele leads to formation of a functional thymus in adult *R/−; CreERt2* mice

To test whether functionally competent TEPC were present in adult *Foxn1^R/^; R26^CreERt2^* (called R/−; CreERt2 herein) mice, 3–4 month old R/−; CreERt2 mice were treated with a single intraperitoneal (IP) injection of 4-hydroxy tamoxifen (4OHT) at different doses, and analyzed seven weeks later for structural and functional changes.

**Figure 1:**
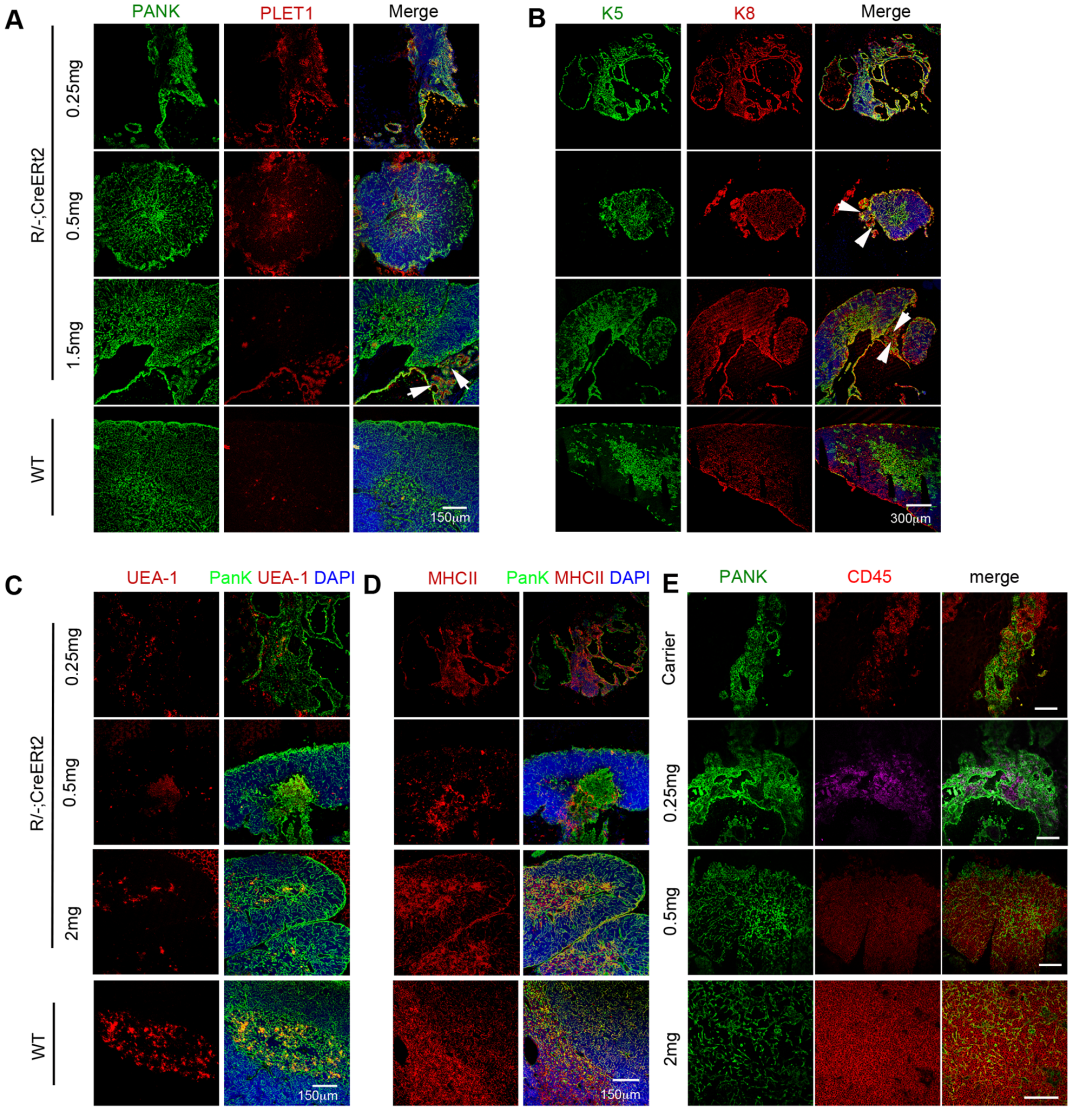
Thymus formation upon Cre-mediated reversion of the *Foxn1^R^* allele in *R/−; CreERt2* mice. 3–4 months old *R/−; CreERt2* mice were injected with 4OHT at the doses shown. Images show immunohistochemical analysis of thymi or thymic rudiments from 4OHT mice 7 weeks post-injection or from a 6 week old C57BL/6 wild type (WT) control. Staining for (**A**) pan-cytokeratin (PANK; green) and PLET1 (red). Scale bars, 150 µm. (**B**) K5 (green) and K8 (red). Scale bars, 300 µm. (**C**) PANK (green) and UEA-1 (red). Scale bars, 150 µm. (**D**) PANK (green) and MHC Class II (MHCII, red). Scale bars, 150 µm. Arrowheads in (**A**) and (**B**) indicate areas of undifferentiated thymic rudiment. DAPI reveals nuclei (blue) in panels (**A–D**). (**E**) PANK (green) and CD45 (red). Scale bars 100 µm. Note that CD45^+^ cells are found associated with but not within the epithelium in thymic rudiments from carrier only and 0.25 µg 4OHT injected mice. 0.25 mg and 0.5 mg 4OHT injected mice and WT controls, n>3. 1.5 mg and 2 mg 4OHT injected mice, n = 1 for each condition; equivalent data were obtained from mice injected with 1.0 mg 4OHT (n = 3).

**Figure 2:**
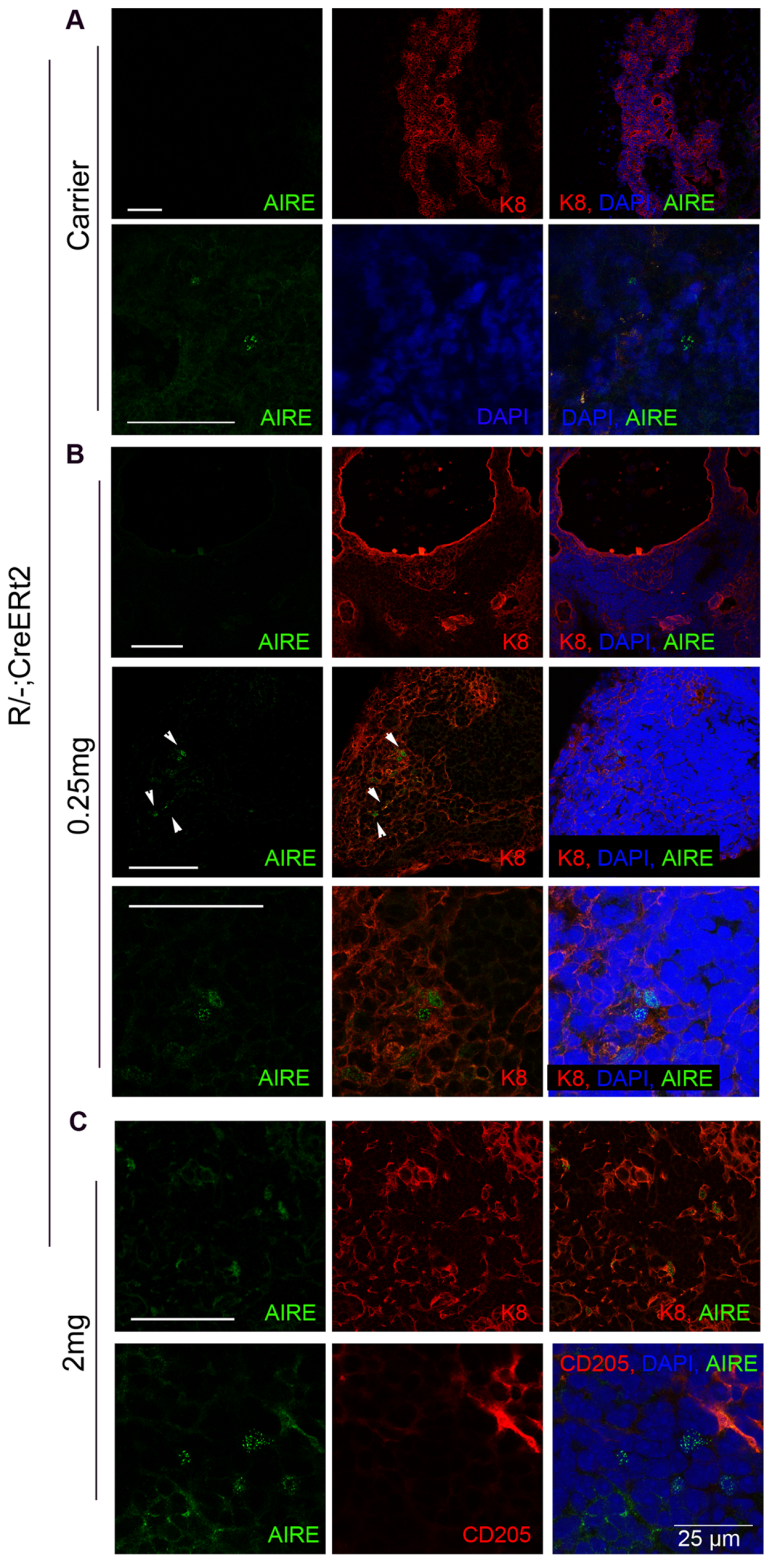
AIRE expression in the unreverted *R/−; CreERt2* thymic rudiment. 3–4 months old *R/−; CreERt2* mice were injected with 4OHT at the doses shown (**A**, carrier-only, **B**, 0.25 mg 4OHT, **C**, 2 mg 4OHT). Images show immunohistochemical analysis of thymi or thymic rudiments from 4OHT mice 7 weeks post-injection. Staining is shown for AIRE, counterstained for cytokeratin 8 (K8) or CD205 as shown. DAPI reveals nuclei (blue). (**A**) Top and bottom panels show representative images of AIRE^−^ regions (which comprised the majority of sections), and a rare AIRE^+^ cell, respectively. (**B**) Top and middle panels show representative images of AIRE^−^ regions and AIRE^+^ cells, respectively. Bottom panel shows higher power image of middle panel. Carrier-only and 0.25 mg 4OHT injected mice, n = 2; reverted mice (i.e. injected with ≥0.5 mg 4OHT), n>3 (shown for 2 mg injected). Scale bars 100 µm except where shown.

**Figure 3:**
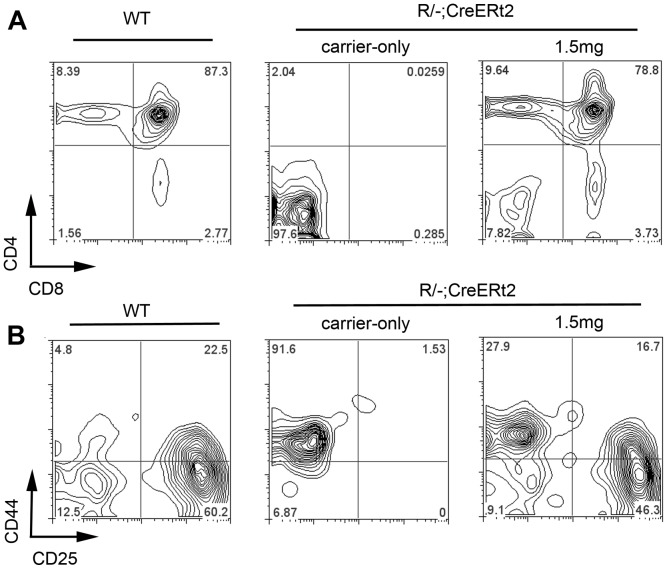
Thymi generated on reversion of the Foxn1 R allele in *R/−; CreERt2* mice support normal T cell development. Thymi from 6 week old C57BL/6 wild type, and *R/− reERt2* mice injected with 4OHT at the doses shown were dissected. Thymocytes were collected and processed for flow cytometric analysis. Plots show staining with the markers shown after gating out dead cells and on CD45^+^ cells. (**A**) Thymi from mice injected with carrier-only contained no CD4^+^ CD8^+^ DP cells (percentage of DP cells: Carrier-only injected, 0.0125±0.015 n = 4; Wild-type, 85.96±1.74 n = 7), while thymi from 1.5 mg 4OHT injected mice contained DP and SP cells. (**B**) Plots show staining with CD44 and CD25 after gating against a lineage cocktail (lin). (lin  = CD3, CD4, CD8, NK1.1, Ter119, CD19, Mac1). Thymic rudiments from carrier-injected mice contained no DN2, DN3 or DN4 thymocytes. The CD45^+^ cells in the DN1 gate most likely represent circulating CD45^+^ cells present in the tissue dissected along with the thymic rudiment, as no CD45^+^ cells were observed within the epithelial component of the rudiment itself; it is possible that these cells might be hematopoietic progenitors attracted by the undifferentiated TEC but not licenced to colonise the epithelial rudiment itself. Thymi from 1.5 mg 4OHT injected *R/−; CreERt2* mice contained all normal DN populations. n>3 for WT and carrier-injected; n = 1 for 1.5 mg (equivalent data were obtained from mice injected with 0.5 mg [n = 3] and 1.0 mg 4OHT [n = 3]).

As previously reported [7], the *R/−; CreERt2* mice were characterized by a small thymic rudiment, with a cystic epithelial structure [7]. This phenotype was also evident in mice injected with 250 µg 4OHT, which showed no evidence of a tamoxifen-induced phenotype (Fig. 1A, B). In this group the thymus rudiment comprised only undifferentiated cystic epithelial cells and no cortical or medullary areas were observed (compare to wild type [WT] In Fig. 1). Cytokeratins 5 and 8 are co-expressed by undifferentiated fetal TEPC but segregate to mark medullary and cortical TEC respectively in the mature thymus [9], [10]. PLET1 marks both the earliest progenitor cells present during thymus organogenesis [10] and most cells in the thymic remnant within adult *nu/nu* mice [11]. Most epithelial cells in mice injected with 250 µg 4OHT co-expressed cytokeratins 5 and 8 (Fig. 1B), and were also positive for PLET1 (Fig. 1 A). UEA-1 staining, which marks only medullary TEC in the adult WT thymus by immunohistochemistry, was detected in a few cells in the un-reverted *R/−; CreERt2* mice (Fig. 1C), consistent with the staining profile in *nu/nu* mice [7]. MHC Class II staining was present throughout the epithelial area in the postnatal thymic rudiment of *R/−; CreERt2* mice and *R/−; CreERt2* mice injected with 250 µg 4OHT (Fig. 1D), as previously observed in fetal *Foxn1^R/−^* mice [7]. However, as previously reported [7], the epithelial component of *R/−; CreERt2* thymi did not become colonized with haematopoietic progenitors and could not support T cell development (Figs. 1E, 2 and 3), and this was also true of *R/−; CreERt2* injected with 250 µg 4OHT (Fig. 1E); although CD45^+^ cells often surrounded the unreactivated epithelial rudiments in both carrier- and 0.25mg 4OHT-injected mice (Fig. 1E) suggesting that, similar to the fetal thymic rudiment, these cells might attract hematopoietic progenitors.

At doses of 0.5 mg of 4OHT and above, a thymic structure comprising both cortical and medullary regions was observed by 7 weeks post-injection (Fig. 1A–D), indicating that upon reversion of the *Foxn1* hypomorphic allele TEPC present in the *R/−; CreERt2* thymic rudiment could generate a functional thymus. These thymi supported normal T cell development, as evidenced by the presence of CD4^+^CD8^+^ double positive (DP) and CD4^+^ and CD8^+^ single positive (SP) thymocytes (shown for 1.5 mg 4OHT in Fig. 3A). Analysis of the double negative (DN) thymocyte population based on CD25 and CD44 staining [12] confirmed the presence of all normal DN subsets in mice that received doses of ≥0.5 mg 4OHT (shown for 1.5 mg 4OHT in Fig. 3B). Furthermore, they contained AIRE*^+^* mTEC (Fig. 2), which are required for induction of central tolerance [13], [14]. Thus, the thymi generated upon reversion of the *Foxn1^R^* allele in this model were functional with respect to capacity to support T cell development. We note that rare AIRE^+^ epithelial cells were present in carrier- and 0.25 mg 4OHT-injected thymi (Fig. 2). These AIRE^+^ cells were always present within linear epithelial aggregates, that were morphologically highly similar to the linear epithelial aggregates found in the *nu/nu* thymus and did not represent areas of mature thymic tissue, indicating that they had arisen by stochastic differentiation of *Foxn1^R/−^* TEPC.

In all *R/−; CreERt2* mice that received 0.5 to 1.5 mg 4OHT, PLET1^+^K5^+^K8^+^ areas of undifferentiated thymic rudiment were present adjacent to the thymus structure (arrowheads, Fig. 1A, B). These PLET1^+^ regions were clearly different from the scattered PLET1^+^ areas normally observed within the medulla of WT thymi, and detected within the medulla formed following *Foxn1* reactivation (Fig. 1A). Indeed, they were histologically and phenotypically similar to the undifferentiated thymic remnants within mice injected with 0.25 mg 4OHT, in which no reversion of *Foxn1^R^* was observed (Fig. 1A), and *nu/nu* mice [11], suggesting they represented non-reactivated TEPCs.

In these experiments, the size of the thymi induced by reversion of *Foxn1^R^* appeared proportional to the dose of 4OHT, with 0.5 mg 4OHT resulting in generation of a small area of thymus tissue, and 1 mg, 1.5 mg and 2 mg 4OHT much larger thymi (shown for 0.5 mg and 1.5 mg in Fig. 1A–D). As reversion of FOXN1 in a single cell is sufficient to generate a small thymus [6], this suggested that at least at higher doses of 4OHT, multiple TEPC were being activated to differentiate. Furthermore, it demonstrated that TEPC were present in *R/−; CreERt2* mice until at least 4 months of age.

### Detection of tamoxifen-independent CreERt2-mediated recombination in *R/−; CreERt2* mice

**Figure 4:**
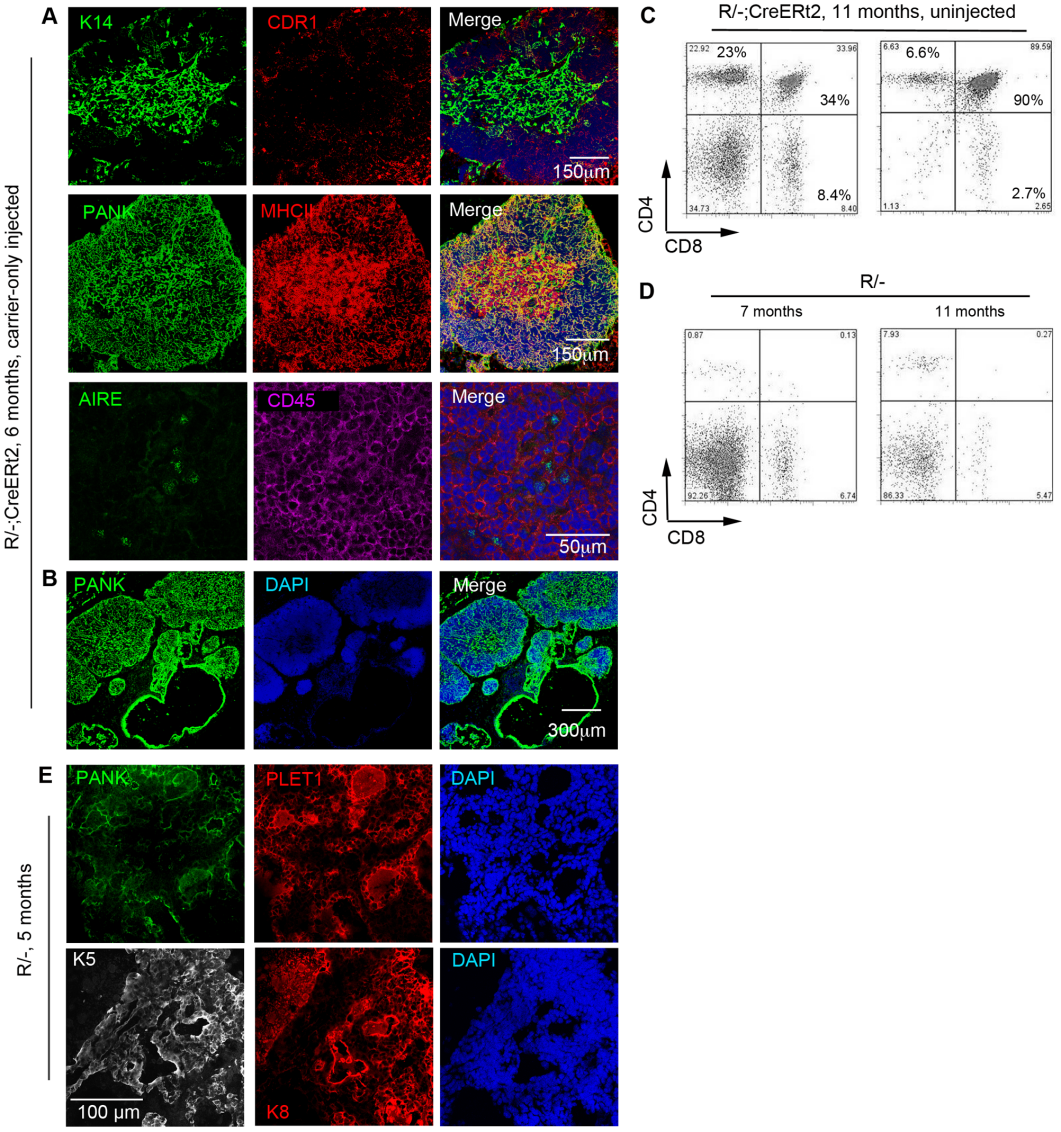
Spontaneous reversion of the R allele in aged *R/-; CreERt2* mice. Images show representative immunohistochemical analysis of carrier-only injected 6 month-old *R/−; CreERt2* mice analyzed one month after injection. (**A**) Carrier-only injected mice exhibited a thymus structure containing both cortical and medullary regions (cytokeratin 14 (K14) and CDR1 indicate medullary and cortical TEC respectively, while pan cytokeratin identifies all TEC). Scale bar: 150 um. **B**, the thymic region of a carrier-only injected mouse, showing a series of small thymic lobes. Scale bar: 300 µm. (**C,D**) Plots show analysis of thymocytes for CD4 and CD8 expression after gating on live CD45^+^ cells for (**C**) two randomly selected, untreated 11 month old *R/−; CreERt2* mice and (**D**) two untreated *R/−* mice (7 m and 11 m old, respectively). Each of the untreated *R/−; CreERt2* mice contained DP cells within the CD45^+^ population (33.9% and 85.6%, respectively) while the *R/−* mice contained no DP cells (mean±SD: Untreated *R/−; CreERt2* mice, 6 months old, 48.20%±40.88%, n = 5; 11 m old, 61.78%±39.34%, n = 2. *R/−* controls 1.09%±1.55%, n = 2). Of note is that the CD4^+^ and CD8^+^ single positive populations present in the aged *R/−* mice are also commonly observed in aged *nu/nu* mice, and are thought to arise by homeostatic expansion of extrathymically-generated T cells [30]. (**E**) Representative images of thymic rudiments from 5 month-old *R/−* mice after immunohistochemical analysis with the markers shown.

Although in the above experiments activity of the CreERt2 fusion protein was strictly tamoxifen-dependent as anticipated, low-level tamoxifen-independent Cre-mediated recombination in CreErt2 strains is well documented (see e.g. [6], [15]). The basis of this is poorly understood, but anecdotally, different sub-strains from individual CreERt2 expressing lines can exhibit strict ligand-dependence or some ligand-independence in their Cre-recombinase activity. Following completion of the experiments described above, we bred the *R/−; CreERt2* line for some months without further functional analysis. During this period, we inadvertently selected a sub-line of *R/−; CreERt2* mice in which tamoxifen-independent Cre-mediated recombination occurred, since reversion of the R allele in *R/−; CreERt2* mice in the absence of 4OHT induction was observed in 6 month-old mice analyzed one year after completion of the experiments documented in Figs. 1 and 2 (Fig. 4A, B). Interestingly, within these reactivated mice, a series of small lobes containing both cortex and medulla was always observed adjacent to the undifferentiated cystic epithelial cells (Fig. 4B), suggesting that multiple recombination events had occurred. Flow cytometric analysis of CD4 and CD8 expression indicated that this reactivated tissue supported T-cell development (Fig. 4C). The possibility that these thymi also arose with time in unreverted *R/−* mice was excluded by comparing the intrathymic CD4 and CD8 staining profile of *R/−; CreERt2* mice and *R/−* mice. While DP cells were detected within untreated aged *R/−; CreERt2* mice, there were no DP cells within aged matched *R/−* controls (Fig. 4C, D). Furthermore, the thymic rudiment of 5 months old *R/−* mice remained unchanged in terms of structure and phenotype compared to young controls, comprising linear aggregates and cystic structures of Plet1^+^K5^+^K8^+^ TEC Fig. 4E). Collectively, these data indicated that the spontaneous recombination observed in *R/−; CreERt2* mice was CreERt2-dependent.

### Accumulation of tamoxifen-independent reversion events with age indicates the long-term existence of an epithelial progenitor cell pool in *R/−; CreERt2* mice

Having established that tamoxifen-independent recombination occurred in some aged *R/−; CreERt2* mice, we set out to test whether this model could provide evidence of continuing activation of TEPC throughout the lifespan. Not all untreated *R/−; CreERt2* mice showed tamoxifen-independent recombination, as indicated by the absence of mature thymus-like tissue and of DP cells. Indeed, the proportion of mice exhibiting evidence of *Foxn1^R^* reversion increased with age (Table 1), indicating that the number of reversion events must accumulate with age. From these data, we concluded that a pool of functional TEPC must exist until at least between 6 and 10 months old in male mice, and between 4 and 6 months old in female mice. Collectively, in this model, tamoxifen-independent recombination occurs until at least 6 months of age and multiple recombination events can occur in each *R/−; CreERt2* thymus.

**Table 1:** Evidence for continued tamoxifen-independent recombination with age in *R/−; CreERt2* mice.

Sex	Recombination status of *R/−; CreERt2* mice	Age				p-value*
		2 months	4 months	6 months	10 months	
**Males**	No recombination	1	1	1	0	
	Recombination	0	6	10	6	
	Recombination frequency (%)	**0/1 (0%)**	**6/7 (85.7%)**	**10/11 (90.9%)**	**6/6 (100%)**	
**Females**	No recombination	1	2	0	0	
	Recombination	1	12	8	1	
	Recombination frequency (%)	**1/2 (50%)**	**12/14 (85.7%)**	**8/8 (100%)**	**1/1 (100%)**	
**Total (males + females)**	No recombination	2	3	1	0	
	Recombination	1	18	18	7	
	Recombination frequency (%)	**1/3 (33.3%)**	**18/21 (85.7%)**	**18/19 (94.7%)**	**7/7 (100%)**	**p = 0.0499**

The table shows the total number of uninjected and carrier-only injected *R/−; CreERt2* mice analyzed within each age group, for male and female mice. Uninjected and carrier-injected *R/−; CreERt2* with equivalent or fewer cells in the DP gate to *R/−* controls were considered not to have undergone tamoxifen-independent Cre-mediated recombination. When the results for males and females are combined, the proportion of mice showing evidence of tamoxifen-independent Cre-mediated recombination varies significantly with age and there is a trend for this to increase with age. *p-value was calculated to compare the recombination frequencies among the four ages by 2×4 Fisher's Exact test.

**Figure 5:**
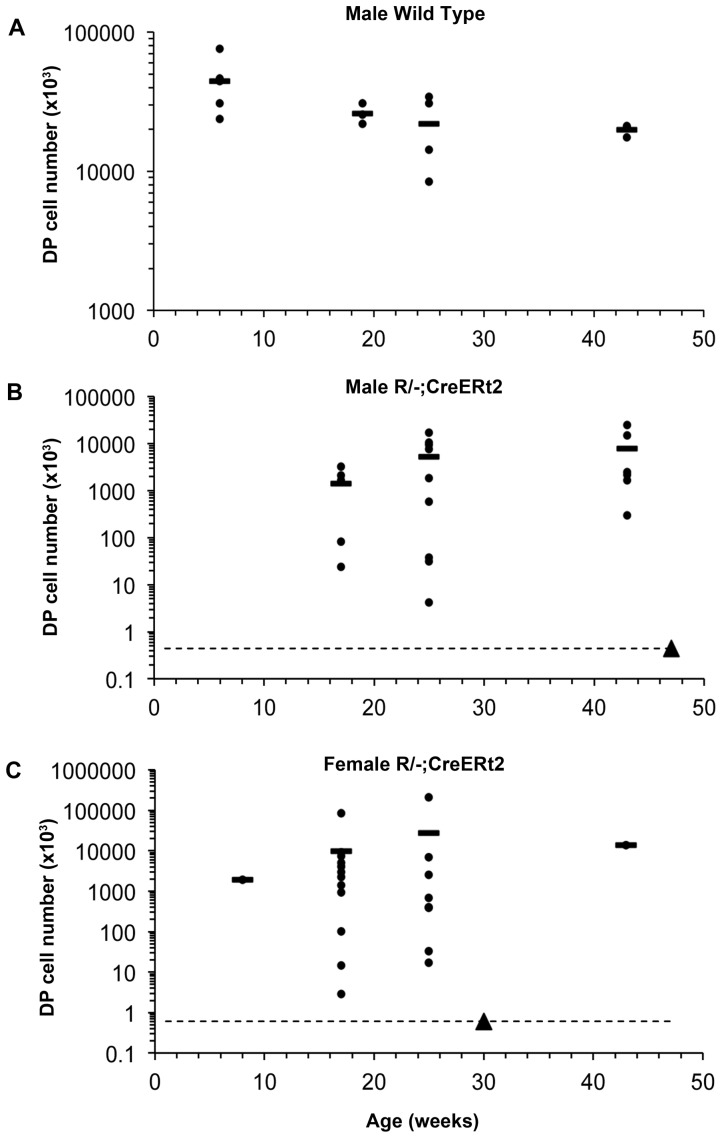
Quantification of DP cell number over time. Thymi from wild type, or uninjected and carrier-injected *R/−; CreERt2* mice of the ages shown were dissected, and thymocyte subset profile was determined by flow cytometric analysis after staining for CD4 and CD8. The number of CD4^+^CD8^+^ DP thymocytes present in each thymus was determined. Graphs show data from (**A**) male wild type C57BL/6 mice; p<0.05 for 6 weeks versus 43 weeks old, (**B**) male uninjected and carrier-injected *R/−; CreERt2* mice and (**C**) female uninjected and carrier-injected *R/−; CreERt2* mice. Each dot represents an individual mouse and the lines show the means. The dotted lines in (**B**) and (**C**) show the number of cells present in the DP gate of *R/−* controls in which Cre recombinase could not be expressed. Closed triangle on all graphs, *R/−* control. Data from uninjected and carrier-injected *R/−; CreERt2* mice with equivalent or fewer cells in the DP gate to *R/−* controls (i.e. on or below the dotted lines) were not included in the analysis presented in Fig. 5, as these mice were considered not to have undergone tamoxifen-independent Cre-mediated recombination (see Table 1).

The WT thymus undergoes a stereotypical age-related involution that results in a progressive loss of thymus size and architecture [16]. Therefore, we next tested whether the thymi generated from the TEPC activated upon reversion of *Foxn1R* also diminished in size over time. The number of DP thymocytes is proportional to the number of functional mature TECs, unless TECs are functionally compromised [7], [17], [18]. Therefore, we analyzed DP cell numbers from all mice showing evidence of *Foxn1^R^* reversion. There were no significant differences in the number of DP cells between carrier-injected and uninjected mice at each age analyzed (not shown), and thus data from uninjected and carrier-injected mice were pooled for analysis. However, data from male and female mice were considered separately due to sex-specific differences observed in the size of the thymi generated upon *Foxn1^R^* reversion (Fig. 5).

As expected, the number of DP thymocytes in wild-type mice declined with age (Fig. 4A). In contrast, there was no age-related decline in DP thymocyte numbers in male or female *R/−; CreERt2* mice. Indeed, for both male and female *R/−; CreERt2* mice there was a trend in the opposite direction (Fig. 5B,C), presumably due either to the growth of TECs generated by tamoxifen-independent recombination in young mice, or to an increase in the number of tamoxifen-independent recombination events and thus activation of additional TEPC with age. As WT C57BL/6 mice undergo age-related thymus involution from 6 weeks after birth (Fig. 5A), we reasoned that any TECs generated in *R/−; CreERt2* by tamoxifen-independent recombination before this age would undergo the same process – and that age-related thymic involution would affect TECs generated at any age after 6 weeks [19]. As there was no evidence for a decline in DP cell numbers with age in *R/−; CreERt2* mice over the 10 months of the study (Fig. 5B,C), we again concluded that un-reactivated *R/−; CreERt2* TEPCs must persist until at least between 6 and 10 months of age *in vivo*, and that *R/−; CreERt2* TEPCs must undergo reactivation events to generate new mature TECs throughout this period. Importantly, although the *Foxn1^R^* allele expresses SV40 T antigen under the control of the *Foxn1* promoter, the SV40 T antigen cDNA is excised upon reversion of the allele [7], so T antigen is no longer expressed in TECs and does not complicate interpretation of these data.

## Discussion

We have shown that fetal TEPC, when unable to express the pivotal pro-differentiative transcription factor FOXN1, can persist *in vivo* for at least 6 months while retaining their capacity to differentiate to produce a functional thymus upon release of the block in normal FOXN1 expression. We further show that the thymi built by continuous activation of TEPC resist the decrease in size associated with age-related thymic involution, as evidenced by their sustained size until at least 10 months of age. Our findings extend current understanding of thymic epithelial cell biology, and raise a number of interesting points.

During thymus organogenesis, TEPC form in the absence of high-level FOXN1 expression. These cells appear to be specified to the TEC lineage by factors acting upstream of *Foxn1*, since *Foxn1* null TEPC express FOXN1-independent TEC lineage markers such as FOXG1 and IL-7, and express transcripts driven from the *Foxn1* promoter. During normal organogenesis however, high-level FOXN1-expression is initiated at E11.25 (with some variation between mouse strains), and all TEC appear to go through a FOXN1-positive stage before graded levels are established in the late fetal and postnatal thymus (manuscript in preparation). Most fetal TEPC will enter the TEC differentiation programme. However, although it is still not clear how postnatal TEC are maintained, the TEPC that function to replenish TEC in the postnatal thymus must either be set aside from this population or arise subsequently by differentiation. Related to this question, several studies have shown that within the fetal thymus, TEC expressing the cell surface protein PLET1 can differentiate to generate both cTEC and mTEC [10], [20]–[22]. This early fetal PLET1^+^ TEC population is sufficient to generate a functional and properly patterned thymus upon transplantation, but the ability of PLET1^+^ TEC to generate a *de novo* thymus appears to be extinguished by E18.5, suggesting that postnatal thymic epithelial stem cells (TESC) either lack this property, or exhibit a different phenotype. However, analysis of a revertible null allele of *Foxn1* has shown that restoration of FOXN1 function in single neonatal *Foxn1* null TEPC (up to postnatal day 14) results in the production of mini-thymi containing cortical and medullary compartments, suggesting that a lineal relationship between fetal and adult TEPC may be possible [6]. Our findings extend these data to show that, if differentiation of early fetal TEPC is blocked by severely limiting the level of *Foxn1* expression, these developmentally arrested TEPC can function as stem cells for at least 6 months *in vivo*, and remain poised to differentiate throughout this period.

In the model described here, we have analyzed thymi produced from cells in which reversion of the *Foxn1^R^* allele has occurred stochastically, in the absence of tamoxifen. By establishing the functionally viability of these *Foxn1^R/−^* TEPC for at least 6 months *in vivo*, we demonstrate the remarkable stability of this cell type. Our findings thus suggest that further exploration of the progenitor/stem cell properties of postnatal PLET1^+^ TEC is warranted. Furthermore, the size of the thymi generated in this model does not decline with age, in contrast to wild-type thymi which undergo a substantial reduction in size by 10 months old as a result of age-related thymic involution. Since the proportion of mice in which no *Foxn1* reversion can be detected decreases over time, we assume that this reflects continued activation of TEPC until at least 6–10 months of age. This finding suggests that exhaustion of TEPC, or changes in TEPC phenotype, with age may contribute to age-related thymic involution. It further suggests that limitations in the haematopoietic progenitor cell pool may not be a major cause of age-related thymic involution [23].

Finally, our data indicate that, although *Foxn1* expression levels in TEC fall with age [24]–[26], reversion of the *Foxn1^R^* allele even in 10 months old mice results in generation of new TEC, indicating that the TESC generated in this model remain poised to express sufficient FOXN1 to support the TEC differentiation programme and generate functional TEC. They therefore suggest that blockade of *Foxn1* expression may be a valuable component of strategies designed to propagate functionally uncompromised TESC *in vitro.*

## Materials and Methods

### Ethics statement

All animal work was conducted according to UK Home Office guidelines, as established in the ANIMALS (SCIENTIFIC PROCEDURES) ACT 1986.

### Mice

*Foxn1^R/−^* mice were generated and maintained as described [7]. *Rosa26^CreERt2/+^* [15] mice were maintained as homozygotes and crossed with *Foxn1^R/−^* mice to generate *Rosa26^CreERt2/+^*; *Foxn1^R/−^* mice as described [7].

### Genotyping

Mice were genotyped as previously described [7].

### Antibodies

MTS24 (IgG2a) [27], a rat mAb that recognises PLET1 [28] was a kind gift from R.L. Boyd; 1D4 (anti-PLET1, rat IgG, [28]), anti-Cytokeratin 8 (Troma 1, rat IgG2a, DSHB); anti-Cytokeratin 14 (AF64, rabbit IgG, Covance)); anti-Cytokeratin 5 (AF138, rabbit IgG, Covance); anti-CD4-FITC or PE (H129.19, rat IgG2a); anti-CD8-FITC (53–6.7, rat IgG2a); anti-CD11b-FITC (M1/70, rat IgG2b); anti-CD19-FITC (1D3, rat IgG2a); anti-CD25-PE (3C7, rat IgG2b); anti-CD44-APC (1M7, rat IgG2b); anti-Ly76-FITC (Ter119, rat IgG2b); anti-CD45-APC (30-F11, rat IgG2b); anti-Cytokeratin (rabbit IgG polyclonal, DAKO); biotinlyated UEA-1 (Lectin, Vector Laboratories); anti-MHC Class II (M5/114.15.2, rat IgG2b, BD Bioscience); anti-AIRE (M-300, SCBT); anti-CD205 (NLDC-145, AbD Serotec); (CDR1 (CDR1, rat IgG2a, Gift from B Kyewski). For detection of unconjugated primaries the following secondary antibodies were used; goat anti-rabbit IgG-alexa488; goat anti-rat IgG-alexa647; Streptavidin-alexa647, goat anti-rat IgG-alexa568 (all Molecular Probes).

### Flow cytometry

Adult thymocytes were isolated by mechanical disruption of dissected thymi and stained with the appropriate antibodies. All staining was for 20 minutes on ice in PBS/5%FCS/5U/ml DNAseI. Data were acquired using FACS Cailibur or LSR Fortessa (BD Biosciences) cytometers and analyzed using Flowjo version 7.1 (Tree Star, Inc) software. For all samples, compensations were determined using antibody capture beads (made in house by S. Monard and O Rodrigues) and Fluorescence Minus One (FMO) staining was used to determine positivity for each antibody. 7AAD or DAPI was used to identify dead cells in all samples.

### Immunohistochemistry

Adult thymus or thymic rudiment tissue was processed for immunohistochemistry as described [29]. Isotype controls (not shown) were included in all experiments. Staining was analyzed using a Leica AOBS confocal microscope (Leica Microsystems GmbH). The images presented are either single optical sections or projected focus stacks of serial optical sections.

### Tamoxifen injection

Mice were treated with a single intraperitoneal injection of 4-hydroxy tamoxifen (4OHT) of the stated dose, prepared in ethanol and diluted appropriately in Cremophor (Sigma)/PBS.

### Intrathymic cell count

Dissected thymus or thymic rudiment tissue was processed as described above. The total number of cells (Nt) were counted. Cells were then stained with CD45, CD4 and CD8 and analyzed by flow cytometry. For analysis in FlowJo, non-viable cells were gated out using DAPI or 7AAD, and the percentage of CD45^+^ cells was recorded (P45). Cells were further analyzed with CD4 and CD8 and the percentage of double positive CD4^+^CD8^+^ cells was recorded (Pdp). The total intrathymic DP cell number was determined as follows, Nt×P45%×Pdp%.

### Statistical analysis

Fisher's Exact test was used to analyse categorical frequency data with an online statistical calculator (http://vassarstats.net/index.html). P value in Fig. 5 calculated using Student's t-test.
